# Case Report: A Rare Orbital Abscess Caused by Dacryocystitis After Administration of Rebamipide Ophthalmic Suspension

**DOI:** 10.3389/fmed.2021.646397

**Published:** 2021-08-10

**Authors:** Hitoshi Imamura, Hiroshi Eguchi, Masuo Sakamoto, Fumika Hotta, Hitoshi Tabuchi, Shunji Kusaka

**Affiliations:** ^1^Department of Ophthalmology, Tsukazaki Hospital, Himeji, Japan; ^2^Department of Ophthalmology, Faculty of Medicine, Kindai University, Osakasayama, Japan; ^3^Department of Technology and Design Thinking for Medicine, Hiroshima University, Hiroshima, Japan

**Keywords:** rebamipide ophthalmic suspension, lacrimal sac concretion, calcium phosphate, dacryocystitis, orbital abscess, case report

## Abstract

**Background:** Rebamipide ophthalmic suspension was launched in Japan in 2012 and is used for the treatment of dry eye.

**Case Presentation:** We report two cases of orbital abscess, which resulted from dacryocystitis that occurred after administration of rebamipide ophthalmic suspension. Computed tomography images showed an eyeball deformity in one case and exophthalmos in the other. In both cases, light microscopy, scanning electron microscopy and energy dispersive X-ray examinations revealed lacrimal sac concretions, which contained calcium phosphate crystals that were surrounded by microorganisms. Lacrimal sac concretion removal from the lacrimal sacs during dacryocystorhinostomies was performed on both patients. Although the postoperative outcome was favorable in one case, vision was lost in the other case due to the development of retinal artery occlusion as a complication of the orbital abscess, despite lacrimal sac concretion removal and administration of antimicrobials.

**Conclusions:** This is the first case report to highlight that rebamipide ophthalmic suspension can cause an orbital abscess via development of lacrimal sac concretion. Ophthalmologists should be aware that rebamipide ophthalmic suspension might induce the formation of concretion in the lacrimal sac.

## Introduction

Rebamipide, (2RS)-2-(4-Chlorobenzoylamino)-3-(2-oxo-1,2-dihydroquinolin-4-yl) propanoic acid, was first launched in Japan in 1990 as an antiulcer agent. Later, it was relaunched in 2012 as an ophthalmic suspension (Mucosta UD 2%), developed by Otsuka Pharmaceutical Co., Ltd. (Tokyo, Japan). It is used for the treatment of dry eye because of its ability to increase the mucin content of the tear film and its anti-inflammatory effects (1, 2). The occurrence rate of dacryocystitis and foreign body in the lacrimal duct after administration of rebamipide ophthalmic suspension administration was recently reported to be 0.02 and 0.04%, respectively (3); however, their aetiologies are uncertain. Herein, we report two rare cases of infectious orbital abscess after administration of rebamipide ophthalmic suspension, which was caused by dacryocystitis following lacrimal sac concretion formation. We also infer the mechanism of lacrimal sac concretion formation by assessing pertinent details of the two cases.

## Case Description

This case report adhered to the ethical principles outlined in the Declaration of Helsinki.

Case 1 was that of a 71-year-old woman who had a history of rheumatoid arthritis and was taking methotrexate, bucillamine, and salazosulfapyridine. She was diagnosed with dry eye by a local physician and prescribed rebamipide ophthalmic suspension to be applied four times a day, which she did for ~4 years until March 2018. In early April 2018, the patient experienced pain and pruritus around her left eye and in mid-April 2018 visited a general practitioner, who prescribed anti-allergic eye drops. Five days later, she experienced severe pain and swelling around her left eye and visited Tsukazaki Hospital for treatment.

**Figure 1:**
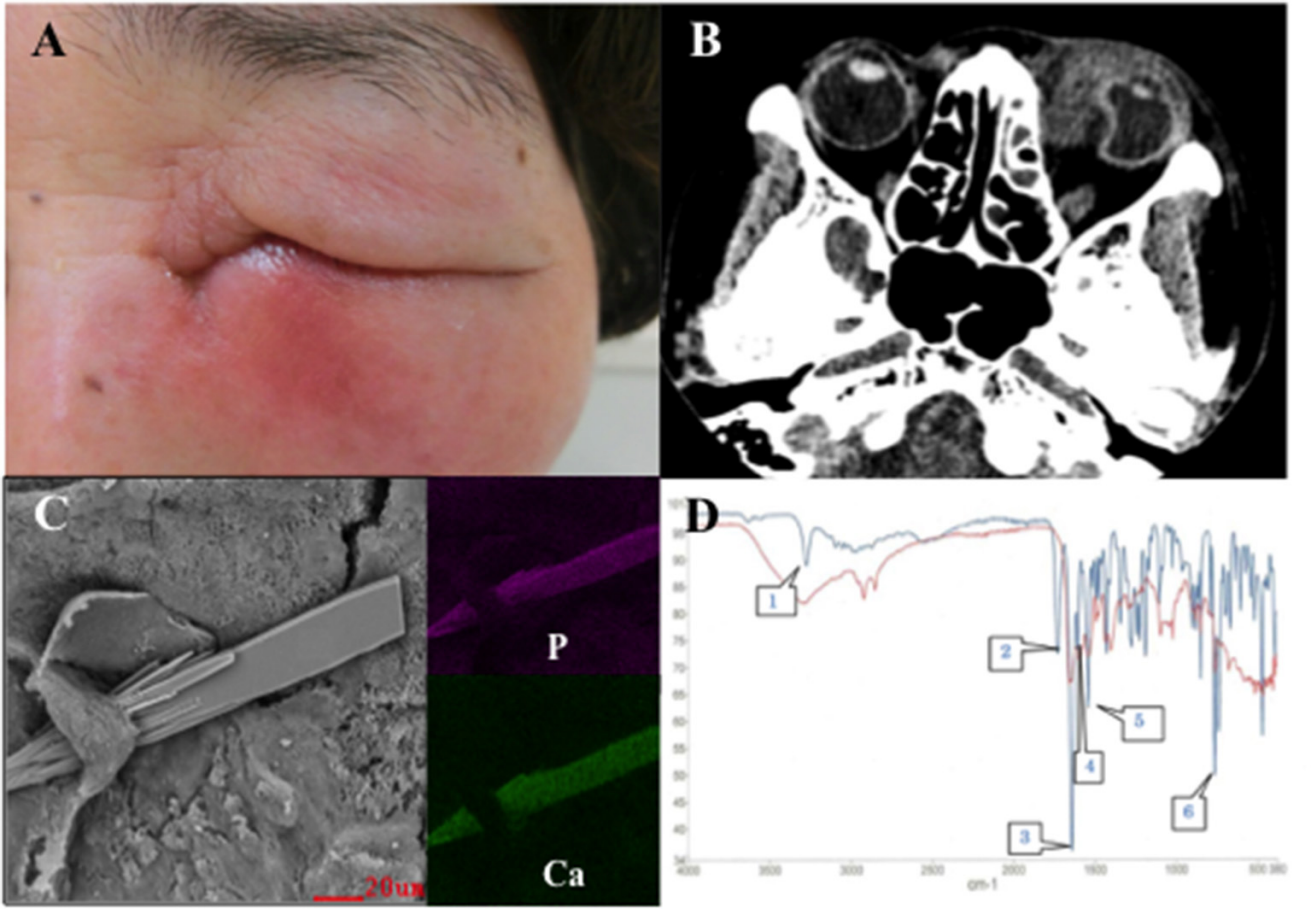
**(A)** Patient's face at the first visit shows redness and swelling around the nasal side of the left lower eyelid. **(B)** Plain computed tomography image shows ocular deformation due to an orbital abscess. **(C)** Scanning electron microscopy with energy dispersive X-ray spectroscopy images of the lacrimal sac concretion removed from the lacrimal sac of the patient in case 1 show sharp structures (left). The structures contain calcium phosphate. Purple indicates the distribution of phosphorus (upper right). Green indicates the distribution of calcium (lower right). **(D)** Infrared spectrophotometry of the lacrimal sac concretion removed from the lacrimal sac of the patient in case 1. The red line denotes the absorption curve of the lacrimal sac concretion and the blue line denotes that of rebamipide. The lacrimal sac concretion has specific infrared absorption bands overlapping with characteristic peaks of rebamipide at 3,280 (speech bubble 1), 1,644 (speech bubble 3), 1,602 (speech bubble 4), 1,540 (speech bubble 5), and 760 (speech bubble 6) cm^−1^.

The patient's visual acuity on the first visit was 20/20 for the right eye; the visual acuity of the left could not be measured because the eyelid was difficult to open. The intraocular pressures were 8 mmHg for the right eye and 13 mmHg for the left eye. Redness and swelling were observed around the nasal side of her left lower eyelid (Figure 1A). Plain computed tomography performed on the same day revealed a high-density mass in the lacrimal sac, ocular cellulitis, and ocular deformation due to an orbital abscess (Figure 1B). On the same day, an incision was made on the lower orbital margin and the intraorbital abscess was drained. *Klebsiella pneumoniae, Streptococcus anginosus, Enterococcus faecalis*, and *Peptoniphilus asaccharolyticus* were isolated from the abscess culture. Daily infusion of ceftriaxone sodium hydrate 1 g was initiated. The next day, her left visual acuity was 20/200. Slight Descemet membrane folds were found in the cornea of the left eye; no inflammation was noted in the eye and the fundus was normal. Computed tomography showed recovery of the left eyeball morphology, but the lacrimal sac concretion was still present.

Two days after her first visit, the vision in her left eye improved to 20/20; the swelling around the eye reduced as well. Three weeks after her first visit, endonasal dacryocystorhinostomy (DCR) was performed on the left side and an 11 × 8 mm lacrimal sac concretion was removed. After the surgery, no symptoms of infection were observed and the postoperative course was uneventful. Light and fluorescence microscopy of the lacrimal sac concretion revealed unstained amorphous crystal-like structures, a high bacterial count, and yeast-like fungi. Scanning electron microscopy with energy dispersive X-ray spectroscopy (SEM-EDX) revealed calcium phosphate crystals with a sharp edge (Figure 1C). Infrared spectrophotometry revealed that the spectra of the lacrimal sac concretion demonstrated specific infrared absorption bands near 3,280, 1,644, 1,602, 1,540, and 760 cm^−1^, which overlapped with the characteristic peaks of rebamipide (Figure 1D). High performance liquid chromatography (HPLC) also revealed that the lacrimal sac concretion had a rebamipide content of 33.3%.

**Figure 2:**
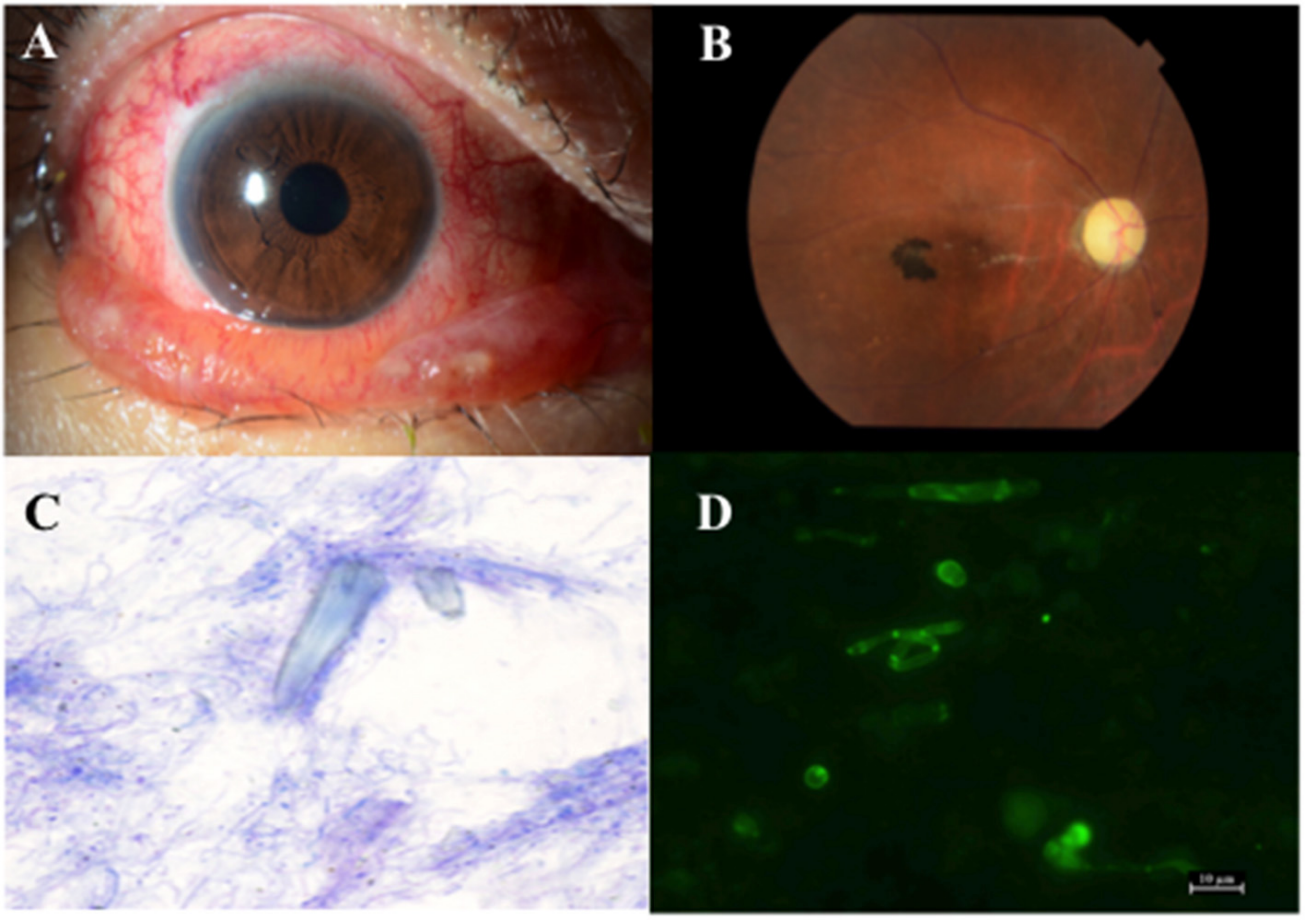
**(A)** Anterior segments of the patient in case 2 show conjunctival hyperaemia and chemosis. **(B)** Fundus photograph of the patient in case 2 shows retinal artery occlusion and optic disc atrophy. **(C)** Microscopic picture of the lacrimal sac concretion after Diff-Quik staining (×1,000) shows bacteria around the amorphous crystal-like structure. **(D)** Microscopic picture of the lacrimal sac concretion after fungiflora Y staining shows yeast-like fungi.

Case 2 was that of a 67-year-old woman, who had a history of rheumatoid arthritis. She was referred to Kindai University Hospital in late April 2018 with complaints of severe pain in the right eye, lid swelling, conjunctival hyperemia, and chemosis (Figure 2A), which had started 7 days prior to her visit. She had been diagnosed with dry eye by a local physician and was prescribed rebamipide ophthalmic suspension to be instilled four times daily, which she did for approximately 2 years.

On initial examination, visual acuities were 20/50 in the right and 20/20 in the left eye. The intraocular pressures were 23 mmHg in the right and 14 mmHg in the left eye. Initially, inflammatory pseudotumor, malignant lymphoma, mucosa-associated lymphoid tissue lymphoma, and IgG4-related diseases were considered as differential diagnoses. After visiting the neuro-ophthalmic clinic to exclude optical diseases, orbital computed tomography was performed 3 weeks after her first visit. Orbital tumor biopsy and abscess drainage were performed because the computed tomography images showed orbital inner wall osteolysis and orbital abscess. Methicillin-resistant *Staphylococcus epidermidis* and quinolone-resistant *Corynebacterium* were isolated from the abscess.

Fifteen days after her first visit, visual acuity of her right eye reduced to 20/1,000 due to retinal artery occlusion (Figure 2B), which occurred even after systemic administration of 0.1 g minocycline daily for 7 days. Four weeks after the first visit, she lost vision in her right eye. Finally, she was diagnosed with retinal artery occlusion due to high intra ocular pressure, which was caused by an orbital abscess that formed after repeated administration of rebamipide ophthalmic suspension. In late June 2018, endonasal DCR was performed on her right side and a 5 × 7 mm lacrimal sac concretion was removed. Light and fluorescence microscopy of the lacrimal sac concretion revealed unstained amorphous crystal-like structures, bacteria (Figure 2C), and yeast-like fungi (Figure 2D). SEM-EDX revealed calcium phosphate crystals. Infrared spectrophotometry of the lacrimal sac concretion showed specific infrared absorption bands near 3,280, 1,644, 1,602, 1,540, and 760 cm^−1^, which overlapped with the characteristic peaks of rebamipide. HPLC revealed that the lacrimal sac concretion had a rebamipide content of >58.6%.

In both cases, the five specific peaks of infrared spectra that were in agreement with those of the rebamipide standard were emitted from the samples, and the retention time in the HPLC analysis of the concretion extract was in agreement with that of the rebamipide standard. These experimental findings indicate the presence of a high concentration of rebamipide in the lacrimal sac concretions.

## Discussion

In this report, we presented the details of two cases of orbital abscess, which resulted from dacryocystitis that occurred after the administration of rebamipide ophthalmic suspension. The pathogenesis of lacrimal sac concretion is unknown; however, several studies have reported that lacrimal sac concretion are comprised of eyelashes (4), epithelial debris (4, 5), granular materials and red blood cells (5). In the present cases, the lacrimal sac concretion contained calcium phosphate crystals and similar components to rebamipide. Both patients in this report had a history of rheumatoid arthritis. Rheumatoid arthritis is a systemic autoimmune disease caused by inflammatory cytokines, such as interleukin-6 (IL-6). IL-6 has been associated with ocular inflammatory diseases such as uveitis (6). In both these cases, chronic inflammation of the lacrimal sac membrane may have resulted in calcium phosphate deposition by a mechanism similar to calcific band keratopathy. Additionally, tear cytokine levels (including IL-6) have been reported to increase in Sjögren syndrome (7).

Rebamipide prevents acute gastric mucosal lesions and accelerates the healing of chronic ulcer. Its known mechanisms of action include the induction of the expression of cyclooxygenase (COX)-2, increases of prostaglandin (PG) E_2_ levels in the gastric mucosa (8), and increases the expression of epidermal growth factor (EGF) and its receptor (EGF-R) (9). It has also been reported to be an oxygen radical scavenger (10).

COX-2 expression is known to be induced by stimulation of cytokines and growth factors (11–13). Given that many collagen diseases overlap in the clinical setting, we speculate that COX-2 was easily induced by a high level of cytokines in the tear fluid and serum in the present cases, in which both patients had a history of rheumatoid arthritis. In addition, rebamipide may have further induced COX-2 and EGF expression in the mucosal epithelium, which in turn could have increased the local concentration of COX-2 in the lacrimal sac. Excessive PG production by COX-2 is involved in exacerbating the inflammatory response, increasing vascular permeability and causing exudative changes. Rebamipide granules may become entangled in the precipitated fibrin, creating a lacrimal sac concretion. Acute dacryocystitis might be caused by the accumulation of microorganisms on the lacrimal sac concretion.

Dacryocystorhinostomy (DCR) may cause difficulty in postoperative ocular surface management in patients with dry eyes, and since both of the patients in this study had a history of tear deficiency dry eye, dacryocystectomy (DC) was considered an alternative treatment method; however, the lacrimal sac concretions in this study were so large that the skin incision would be greatly expanded if DC or external DCR was performed. The lacrimal sac concretion could be removed by endonasal DCR unedr CT evaluation. Moreover, since the lacrimal canal would be wide open to the nasal cavity following endoscopic DCR, exudative materials were drained without the use of drain device, such as the venous catheter (14), which would further contribute to the postoperative anti-inflammation in the lacrimal sac. Postoperative punctal plug or closure could be performed, if necessary. Considering the above-mentioned reasons, we did not opt to perform a DC or external DCR. Nowadays, computer-assisted navigation (CAN) is occasionally used during endoscopic sinus surgery. It is reportedly useful in complex cases wherein there is a risk of compromising the medial rectus or the anterior ethmoidal artery (15). In the current cases, the nasal structure was anatomically normal and the location of the lacrimal canal was confirmed using preoperative CT. Therefore, we were able to perform the surgery without using CAN.

There are certain limitations of this case report. We presented only two cases. However, in Japan, there could potentially be more unreported cases of rebamipide ophthalmic suspension-induced dacryocystitis. The history of rheumatoid arthritis noted in case 2 was merely a self–reported diagnosis. We were unable to measure tear cytokine levels for both of these cases. We have not strictly excluded chronic diseases other than rheumatoid arthritis, which was found in the medical interview, and anemia and diabetes, which were examined in the preoperative blood tests. Therefore, we cannot completely deny the possibility that certain other chronic diseases and the history of medication, including eye drops, could have affected the lacrimal sac concretion development. Further research involving a large number of patients with rebamipide ophthalmic suspension-induced dacryocystitis is needed to elucidate the pathogenesis of the formation of lacrimal sac concretion.

In conclusion, we presented two cases of orbital abscess, which resulted from dacryocystitis that occurred after administration of rebamipide ophthalmic suspension. Ophthalmologists should be aware of the risk of lacrimal sac concretion formation induced by rebamipide ophthalmic suspension, which can cause severe dacryocystitis. If symptoms of dacryocystitis occur in patients using rebamipide eye drops, they should be referred to a hospital specializing in the field of oculoplastic and nasal surgery as soon as possible.

## Meeting Presentation

The details of case 1 were presented at the 72nd annual congress of Japan Clinical Ophthalmology in Tokyo on 11th October 2018.

## Data Availability Statement

The original contributions presented in the study are included in the article/supplementary material, further inquiries can be directed to the corresponding author/s.

## Ethics Statement

Written informed consent was obtained from the patients for publication of this case report and accompanying images.

## Author Contributions

HI and HT made substantial contributions to treating case 1. HE made contributions to conception and design of the case reviews and drafting and revising the manuscript. MS made substantial contributions to treating case 2. FH made substantial contributions to perform microscopic examinations of clinical samples obtained from the two cases. SK made substantial contributions to revising the draft. All authors have read and approved the final manuscript.

## Conflict of Interest

HE has a consultant contract with Otsuka Pharmaceutical Co., Ltd. The remaining authors declare that the research was conducted in the absence of any commercial or financial relationships that could be construed as a potential conflict of interest.

## Publisher's Note

All claims expressed in this article are solely those of the authors and do not necessarily represent those of their affiliated organizations, or those of the publisher, the editors and the reviewers. Any product that may be evaluated in this article, or claim that may be made by its manufacturer, is not guaranteed or endorsed by the publisher.

## Abbreviations

COX: cyclooxygenase
IL-6: interleukin-6
PG: prostaglandin
EGF: epidermal growth factor
DCR: dacryocystorhinostomy
SEM-EDX: scanning electron microscopy with energy dispersive X-ray spectroscopy
HPLC: high performance liquid chromatography.
